# Cattail-Grass-Derived Porous Carbon as High-Capacity Anode Material for Li-Ion Batteries

**DOI:** 10.3390/molecules28114427

**Published:** 2023-05-29

**Authors:** Hui Li, Lingyue Song, Dongxing Huo, Yu Yang, Ning Zhang, Jinglong Liang

**Affiliations:** 1 Key Laboratory of Modern Metallurgical Technology, Ministry of Education, College of Metallurgy and Energy, North China University of Science and Technology, Tangshan 063210, China; 2 College of Mechanical Engineering, North China University of Science and Technology, Tangshan 063210, China; 3 Comprehensive Test and Analysis Center, North China University of Science and Technology, Tangshan 063210, China

**Keywords:** cattail grass, biomass, lithium-ion batteries, energy storage, anode material

## Abstract

Cattail-grass-derived porous carbon as high-capacity anode materials were prepared via high-temperature carbonization and activation with KOH. The samples exhibited different structures and morphologies with increasing treatment time. It was found that the cattail grass with activation treatment—1 (CGA-1) sample obtained at 800 °C for 1 h presented excellent electrochemical performance. As an anode material for lithium-ion batteries, CGA-1 showed a high charge–discharge capacity of 814.7 mAh g^−1^ at the current density of 0.1 A g^−1^ after 400 cycles, which suggests that it has a great potential for energy storage.

## 1. Introduction

With the consumption of energy resources increasing, the demand for energy storage devices is growing [1]. Lithium-ion batteries (LIBs), a very promising energy storage device, have been widely used in daily life and industrial production with many advantages, such as high energy density, long cycle life, and light weight [2,3,4]. For LIBs, electrode materials play a vital role in improving performance. The energy storage capacity of electrode materials is closely related to the performance of the battery [5]. Normally, graphite is used as the anode material for LIBs, but the theoretical capacity of graphite for LIBs is only 372 mAh g^−1^, which cannot satisfy the demand for large-capacity storage of electrical energy [6,7,8]. In order to further improve the energy density of LIBs, it is urgent to explore electrode materials with high energy storage density [9].

Carbon-based anode materials, such as hard carbon [10], carbon nanotubes [11], carbon nanospheres [12], and activated porous carbon [13], are excellent electrode materials with high energy storage density [14,15,16]. In order to obtain high-performance amorphous carbon, there are two methods to use: heteroatom doping and activation treatment. Heteroatom doping refers to modifying carbon material with other kinds of atoms (nitrogen, sulfur, phosphorus) [17], which can increase the distances of graphene layers to improve the storage and transport of lithium ions [18]. Another method is activation treatment, in which the activator reacts with the carbon material to generate a large number of pores and defect structures in the carbon material, thus enhancing the energy storage capacity [19]. Many activators were used in the activation treatment, such as alkali (KOH, NaOH) [20,21], acid (H_3_PO_4_) [22], metal salts (MgCl_2_, CaCl_2_, ZnCl_2_, CuCl_2_) [23,24], and alkali metal salts (K_2_CO_3_, Na_2_CO_3_, K_2_FeO_4_) [5,25], etc. T. Rasheed et al. prepared activated porous carbon with natural walnut shells and K_2_FeO_4_, in which the discharge capacity reached 1220.22 mAh g^−1^ at the first charge–discharge cycle [5]. G. Murali et al. pre-carbonized the peanut shells and activated them with KOH, which has a high density of 680 mAh g^−1^ after 100 cycles at a current of 0.05 C [13]. Activation treatment is an effective method for improving the energy storage capacity of biomass.

Biomass is considered to be one of the most abundant renewable resources on the planet and has extremely high value [26]. Carbon is the main component of biomass, with huge potential in development [27]. Many porous carbon materials derived from biomass have been prepared for LIBs [28,29,30,31]. Due to their high porosity, large specific surface area, and high reversible capacity, carbon materials derived from biomass can replace graphite in traditional LIBs, thereby increasing their energy density [32]. However, the specific capacity of carbon materials that are produced by direct carbonization is low.

Cattail grass is a common plant with a fibrous structure that has potential for preparing porous carbon to produce high-capacity materials. In this work, we propose a new and efficient way to use cattail grass. Cattail-grass-derived carbon was prepared by activation treatment with KOH, which has a large specific surface area and porosity. The cattail grass fleece has a large specific surface area and generates a large number of pores during the preparation of the derived carbon, which enables it to have excellent electrochemical properties. After being experimentally verified, the best sample shows a high reversible capacity after 400 cycles. This provides a basis for improving the utilization of biomass materials and increasing the energy density of lithium-ion batteries. This study demonstrates that cattail-grass-derived carbon can be used as a potential anode material for LIBs.

## 2. Results and Discussion

**Figure 1:**
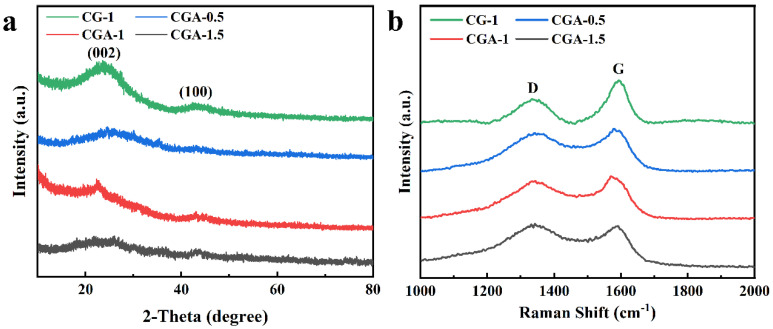
(**a**) XRD patterns and (**b**) Raman spectra of CG-1, CGA-0.5, CGA-1, and CGA-1.5.

The XRD patterns of CG-1, CGA-0.5, CGA-1, and CGA-1.5 are shown in Figure 1a. There were two broad, weak peaks detected at 23° and 43°, which are attributed to (002) and (100) reflections, respectively. The (002) peak suggests that there was parallel stacking of graphene sheets inside the material, while the (100) peak was caused by the sp^2^ hybrid carbon. The presence of these two peaks indicates the formation of an amorphous structure [33]. The peak intensity of the CG-1 samples at 23° was stronger than that of the others, which means that the addition of the activator KOH promoted the formation of amorphous carbon. The peak (002) in the figure shows that the amorphous carbon material contained some fine graphite crystallites. These graphite crystallites were formed by stacking several layers of graphene sheets. The distance of the graphene sheets (*d*_002_) and the thickness of the graphite crystallites (L) can be calculated by Bragg’s law (Equation (1)) and Scherrer’s formula (Equation (2)), respectively, as follows [34]:

(1) $$2d_{002}\sin\theta =\lambda$$

(2) LB⁡cos⁡θ=kλ

where *d*_002_ is the graphene layer distance (nm), *θ* is the Bragg diffraction angle (°), λ is the X-ray wavelength (0.154 nm), *L* is the average thickness of the crystal grain perpendicular to the crystal plane (nm), B is the half-height width of the diffraction peak of the measured sample (rad), and K is Scherrer constant. K is 0.89 when B is the half-height width of the diffraction peak.

The interlayer distance and the thickness of the graphite crystallites in the samples are shown in Table 1. As the amount of activator increased, the interlayer distances gradually increased owing to the cation intercalations [35,36], which were over 0.39 nm. This was beneficial for the insertion of lithium ions and increasing the reversible capacity of the material. According to the Scherrer formula (Equation (2)), it was calculated that the graphite crystallites in the material without being activated contained four layers of graphene stacked, while three layers of graphene stacked were in the activated samples. The results indicate that the activation can decrease the size of graphite crystallites, reduce the degree of graphitization, and increase the degree of disorder.

In order to investigate the disorders of the samples, Raman spectra were collected, as shown in Figure 1b. There were two peaks, located at 1340 cm^−1^ and 1590 cm^−1^, corresponding to the D peak and the G peak, respectively. The D peak is attributed to the characteristics of sp^3^ hybrid carbon, and the G peak is related to the sp^2^ carbon-type structure [37]. In general, the intensity ratio of the D peak and G peak (I_D_/I_G_) can be used to judge the disorders of the materials. The calculation results are shown in Table 1. The value of I_D_/I_G_ of CG-1, without activation treatment, had a lower disorder degree, while the I_D_/I_G_ of CGA-0.5, CGA-1, and CGA-1.5, with activation treatment, had a great increase. It is indicated that the addition of an activator can increase the disorder of the material, which has a great effect on ion transport and storage.

**Figure 2:**
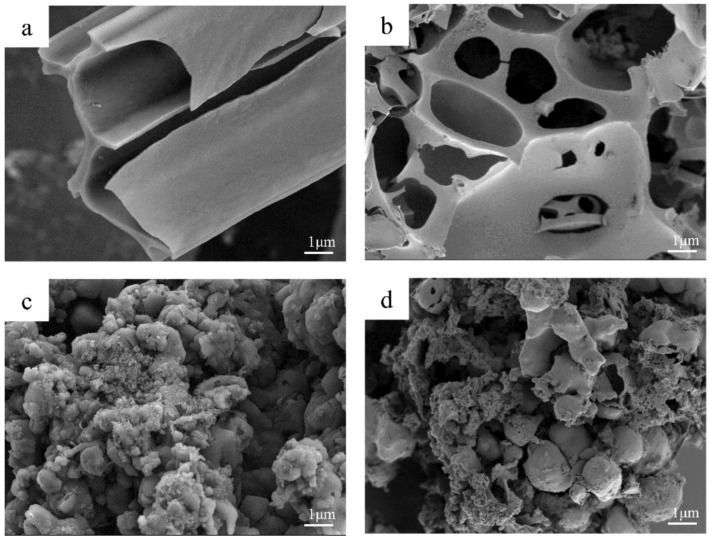
SEM images of (**a**) CG-1, (**b**) CGA-0.5, (**c**) CGA-1, and (**d**) CGA-1.5.

The microstructure and morphology of the samples were characterized using SEM, as shown in Figure 2. Figure 2a is the image of CG-1 without activation treatment. It can be observed that cattail grass fluff still maintained its fibrous structure, while the other samples added with KOH were damaged to various degrees, and the amount of activator enhanced the degree of fragmentation. Compared with CG-1, CGA-0.5 presented some larger pores and defect structures, as shown in Figure 2b, which increased the specific surface area and provided more channels for ion transport. From Figure 2c, it can be seen that the surface of CGA-1 was corroded, with increasing roughness and many fragmentations appearing on the surface. The degree of damage to CGA-1.5 was further increased, as shown in Figure 2d, which formed more pore structures. It is worth noting that activation treatment can change the morphology and structure of the samples to create more pores and defect structures, which is beneficial to improving the performance of the LIBs.

In order to further study the specific surface area, pore structure, and pore size distribution of the material, samples were tested through nitrogen adsorption–desorption isotherms, which are shown in Figure S1a. The isotherms of CGA-0.5, CGA-1, and CGA-1.5 belonged to type IV, indicating the material contained a large number of pore structures. However, the isotherm of CG-1 belonged to type III, and the adsorption capacity was low, which suggests that the internal pores of the material were fewer. Micropore distribution was analyzed using the HK model, as shown in Figure S1b, while mesopore and macropore distribution was calculated using the BJH model in Figure S1c. The data for specific surface area and pore volume are shown in Table 1. The specific surface area (S_BET_) of CG-1 was much lower than that of the other samples, which was only 1.175 m^2^ g^−1^, while the specific surface areas of CGA-0.5, CGA-1, and CGA-1.5 were 886.472 m^2^ g^−1^, 714.808 m^2^ g^−1^, and 755.076 m^2^ g^−1^, respectively. Evidently, the number of pores has a great influence on the change in specific surface area. From the pore volume data, it can be found that CG-1 was also much lower than the activated samples. The volume of mesopores and macropores of CGA-0.5, CGA-1, and CGA-1.5 increased with the prolongation of the reaction time but the volume of micropores did not decrease accordingly. The micropore volume inside of CGA-1.5 was higher than that of CGA-1. The formation mechanism of the pore structure is shown in Figure S1. At first, the carbon material reacted with the activator to generate a large number of micropores. With the reaction time prolonging, the diameter of the micropores became gradually larger. Many micropores aggregated to form mesopores and macropores. Meanwhile, a large number of micropores would still be regenerated. Therefore, the volume of mesopores and macropores in the samples gradually increased with the extension of the reaction time, while the volume of the micropores did not have a close relationship with the reaction time. The porous structure provided more space for lithium ion storage.

**Figure 3:**
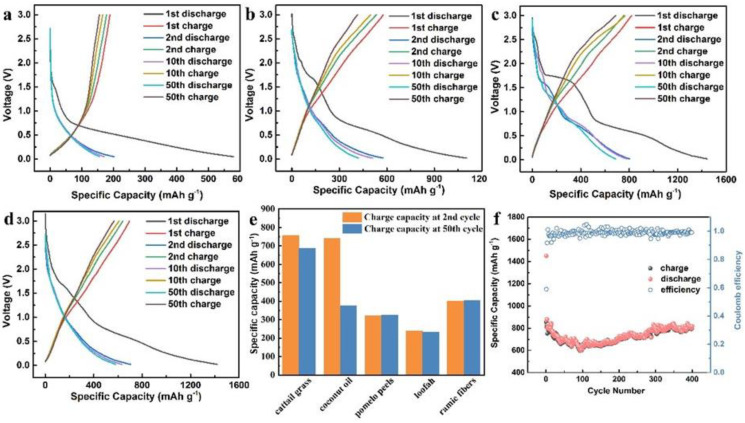
The charge–discharge curves of (**a**) CG–1, (**b**) CGA–0.5, (**c**) CGA–1, and (**d**) CGA–1.5; (**e**) comparison of cattail grass with the state-of-the-art LIBs; and (**f**) cycling performance of the CGA-1 at a current density of 0.1 A g^−1^ in LIBs.

The charge–discharge performance of the samples was analyzed at a current density of 0.1 A g^−1^, as shown in Figure 3a–d. The first discharge specific capacities of the samples were 578.6 mAh g^−1^, 1102.4 mAh g^−1^, 1443.81 mAh g^−1^, and 1420.3 mAh g^−1^, respectively. Obviously, the first discharge specific capacity of the materials increased greatly with the addition of activator. Combined with the results of the nitrogen adsorption test, it can be seen that the activator reacted violently with the precursor to generate more pores and defect structures, which facilitated the storage and transportation of lithium ions. However, the first charge specific capacity of the material was much lower than the discharge specific capacity, which can be attributed to three reasons, as follows: (1) during the first discharge, the electrolyte reacted with the surface of the carbon material to form a solid electrolyte interface (SEI) film, which affected the capacity of energy storage; (2) lithium ions were irreversibly intercalated into some special structure of carbon material, such as some small micropores and defects; (3) the material changed from its initial form to an active lithium storage carbon electrode after the first discharge [37,38]. As the number of cycles increased, the reversible specific capacity of the material gradually decreased. At the 50th cycle, the reversible specific capacities of the samples were 155.5 mAh g^−1^, 415 mAh g^−1^, 688.9 mAh g^−1^, and 568.1 mAh g^−1^, respectively. And the reversible specific capacities of the samples with activation treatment were higher than the theoretical reversible specific capacity of graphite. Among them, CGA-1 exhibited better electrochemical performance, for which the reversible specific capacity was higher than that of other biomass-derived carbon, as shown in Figure 3e [2,4,6,39]. It is indicated that the cattail-derived carbon has great potential for LIBs. Figure S5 shows the cycling performance curves of CG-1, CGA-0.5, CGA-1, and CGA-1.5 at a current density of 0.1 A g^−1^. The CGA-1 sample had the highest specific capacity and better cycling performance. In addition, we performed 400 charge–discharge tests on the CGA-1 and observed its capacity change. The result is shown in Figure 3f. As the number of cycles increased, the sample capacity gradually decreased. After 100 cycles, the material capacity gradually increased. Through many cycles, the active sites on the surface of the material were activated, which led to a gradual increase in the material capacity. After 400 cycles, the reversible capacity of the material was 814.7 mAh g^−1^. As shown in Figure S4, the AC impedance spectra of CGA-1 sample were compared before and after 400 cycles. Before and after cycling, the shape of the AC impedance spectrum remained unchanged; only the reaction impedance increased, further proving that the electrode has excellent cycling stability.

**Figure 4:**
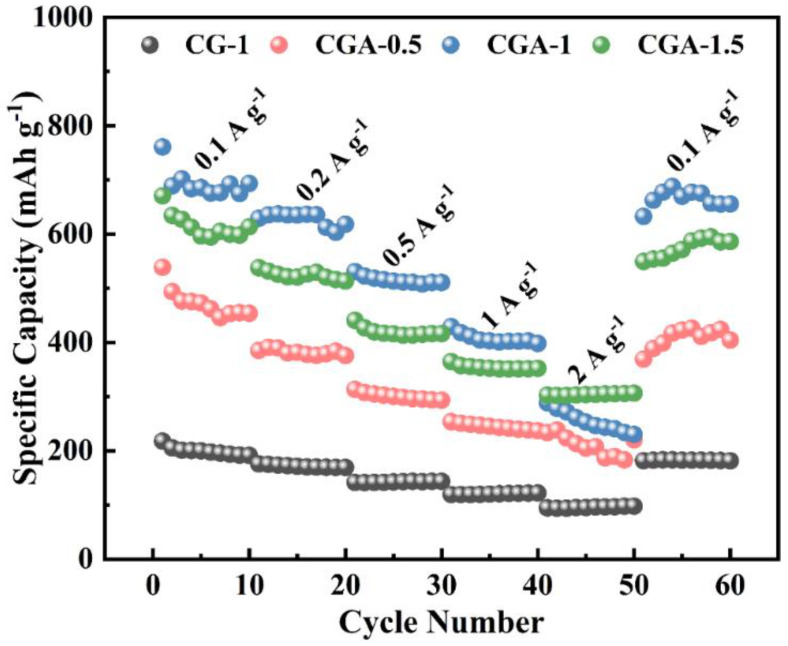
Rate performance of CG–1, CGA–0.5, CGA–1, and CGA–1.5.

The rate performance of the samples is shown in Figure 4 at the current densities of 0.1 A g^−1^, 0.2 A g^−1^, 0.5 A g^−1^, 1 A g^−1^, 2 A g^−1^, and 0.1 A g^−1^. With the increase in current density, the reversible capacity of the samples decreased to varying degrees. When the current density returned to 0.1 A g^−1^, the reversible capacities of the samples reverted back to their initial capacities, which shows that these samples have excellent cycle stability.

**Figure 5:**
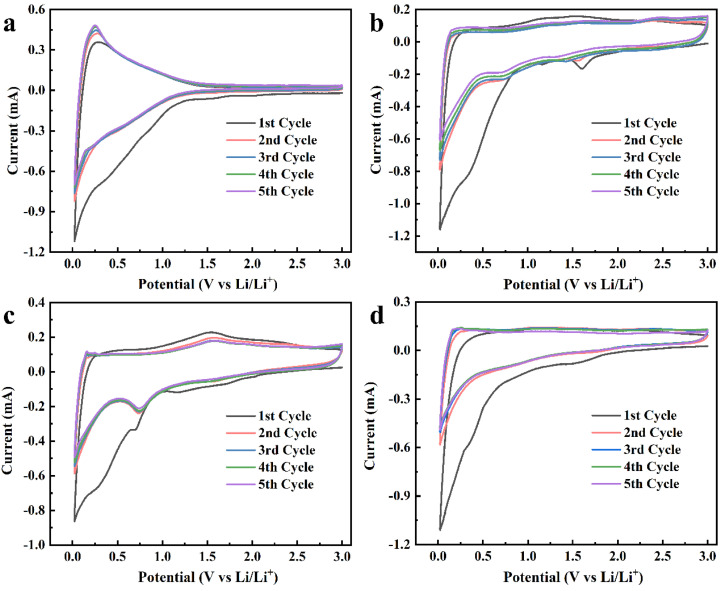
CV curve of (**a**) CG–1, (**b**) CGA–0.5, (**c**) CGA–1, and (**d**) CGA–1.5.

Figure 5 shows the CV plots at a scan rate of 0.5 mV s^−1^ at 0.02~3 V of the samples. In the first cycle, it can be seen that a small reduction peak appeared around 0.3 V, corresponding to the reaction of the electrode material surface with the electrolyte to form an SEI film [39]. In the subsequent cycles, the peak disappeared, which means that the SEI film had formed at the first cycle. In addition, there was another reduction peak near 1.5 V in the CV curves of CGA-0.5, CGA-1, and CGA-1.5, which was caused by the irreversible reaction of lithium ions and materials [7]. In the subsequent cycles, the peak disappeared, and the CV curves had a good degree of coincidence, which reveals that the materials have excellent cycle stability. In five cycles, the CV curves of CGA-0.5, CGA-1, and CGA-1.5 had reduction peaks near 0.7 V with different intensities, among which the peak of CGA-1 was the most obvious; this may be due to the redox reaction between the functional groups of the materials and lithium ions.

**Figure 6:**
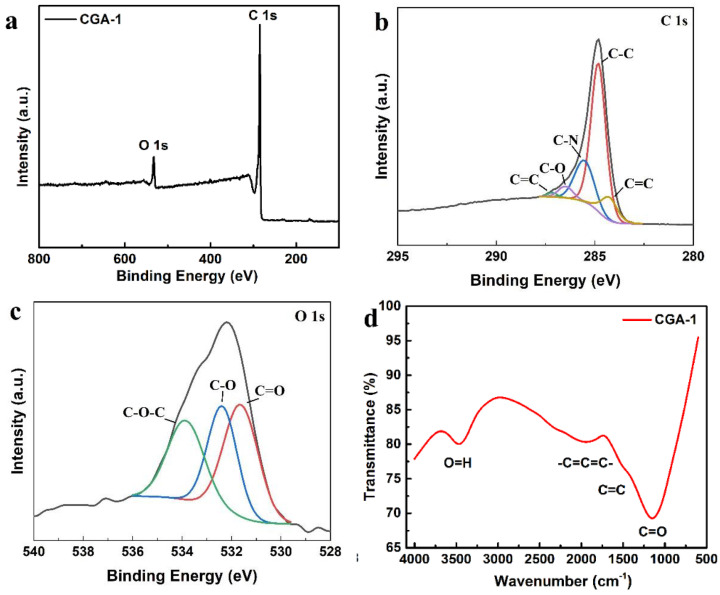
(**a**) XPS full spectra, (**b**) C1s, and (**c**) O1s, and (**d**) IR spectrum of CGA–1.

To further investigate the functional groups contained in CGA-1, XPS and IR were carried out, as shown in Figure 6. Figure 6a is the full-spectrum analysis diagram of CGA-1. There are two peaks centered at 284 eV and 533 eV, corresponding to the C 1s peak and the O 1s peak, respectively, indicating that the material contained mainly carbon and oxygen. The C 1s peak in Figure 6b can be split into five peaks of 284.4 eV, 284.8 eV, 285.6 eV, 286.5 eV, and 287.2 eV, which were assigned to the C=C, C-C, C-N, C-O, and C=O functional groups, respectively. The different chemical states of oxygen can be determined by the O 1s peak in Figure 6c, where three peaks located at 531.7 eV, 532.4 eV, and 533.7 eV correspond to C=O, O-C, and C-O-C functional groups, respectively. Figure 6d is the infrared spectrum of CGA-1. It can be seen that the material contained C=O, C=C, -C=C=C-, and O-H functional groups, which agrees well with the XPS results. The sample contained a large amount of carbon and oxygen, and the main functional groups were C=C, C-C, C-O, and C=O. These functional groups are conducive to forming chemical bonds with lithium ions to enhance energy storage. In addition, no other significant absorption peaks were observed, indicating the absence of additional valence bonds forming between the prepared electrodes. In this case, the results are in agreement with the XRD pattern, confirming the successful preparation of the derived carbon electrodes.

**Figure 7:**
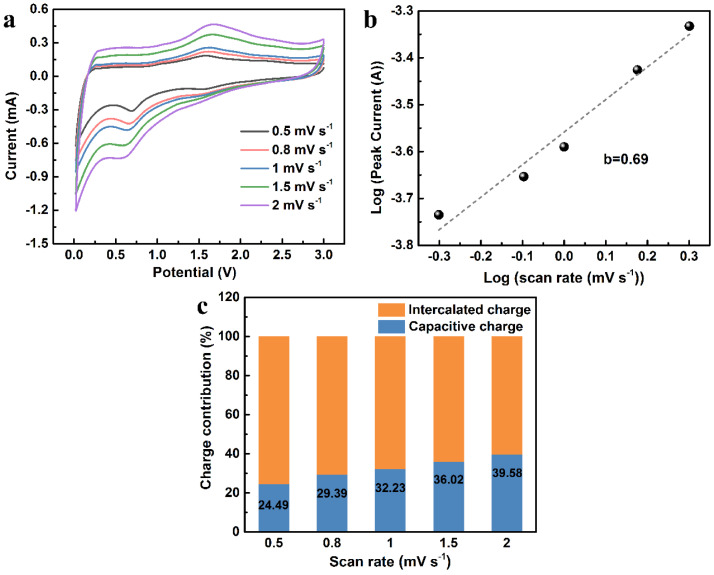
The capacitive contribution calculation of CGA–1. (**a**) CV curves at various scan rates from 0.5 mV s^−1^ to 2.0 mV s^−1^ for CGA–1. (**b**) The linear relation of peak currents and scan rates. (**c**) Capacitive contribution of charge storage at 0.5 mV s^−1^ to 2 mV s^−1^ scan rates.

To further demonstrate the storage mechanism of the CGA-1, quantitative analysis of CV at different scan rates from 0.5 mV s^−1^ to 2.0 mV s^−1^ was performed based on Dunn’s test, as shown in Figure 7a. The scan rate (v) and peak current (i) obey Equation (3), where a and b are variable parameters. Notably, b is related to the intrinsic reaction kinetics of the electrodes, in which a b value of 0.5 demonstrates that the current is contributed by ion diffusion control, and a b value of 1 represents that the current is fully dominated by the capacitive behavior. In Figure 7b, the b value was calculated to be 0.69 between 0.5 and 1, which illustrates the mixed mechanisms in the charge storage process. Furthermore, the current response (i) measured at a fixed voltage can be distinguished into two parts of the contribution through Equation (4), where k_1_*v* represents the contribution of the capacitive process and k_2_*v*^1/2^ corresponds to the process of diffusion control. At different scanning rates from 0.5 mV s^−1^ to 2.0 mV s^−1^, the capacitive charge contribution of the CGA-1 is shown in Figure 7c. A slow scanning rate is beneficial to the full contribution of the diffusion-controlled process of connecting the redox peaks [1]. With the increase in scan rate, the diffusion contribution decreased and the capacitance contribution increased, which demonstrates that the porous structures and multi-functional groups result in fast charge transfer and facilitate the adsorption of ions [37].

(3) $$i=a\nu^{b}$$

(4) $$i\left(V\right)=k_{1}\nu +k_{2}\nu^{1/2}$$

To gain insight into the storage mechanism of lithium ions in the materials, EIS were analyzed at open circuit potential, as shown in Figure S3. The diameter of the semicircle in the high-frequency region determines the internal resistance and charge transfer resistance of the sample, while the straight line of the plot at low frequency corresponds to lithium ion diffusion [40,41]. The CGA-1 with a smaller semicircle and a greater slope of the inclined line implies that the sample had lower resistance and higher lithium ion diffusion rates. The equivalent circuit diagram is shown in Figure S3b. From analyzing the impedance data, CGA-1.5 was determined to have the lowest Rct (172 Ω) and CA-1 had the highest Rct (615 Ω). This proves that CGA-1.5 had good electrochemical reaction properties. Moreover, the impedance values of the samples with activation treatment were much lower than that for CG-1, which indicates that the activation treatment can greatly improve the electrochemical performance of carbon materials.

Based on the above analysis, compared with other previously prepared derived carbon materials from biomass, the derived carbon material prepared from cattail grass in this paper has certain advantages in terms of electrochemical properties, such as specific capacity and charge/discharge cycle stability. Therefore, the research in this paper provides an effective way to develop low-cost and high-performance derived carbon materials. However, in order to achieve commercial applications, the electrode structure and electrochemical properties still need to be optimized to further enhance the cycling stability of the electrode.

## 3. Experimental and Methods

### 3.1. Material Preparation

**Figure 8:**
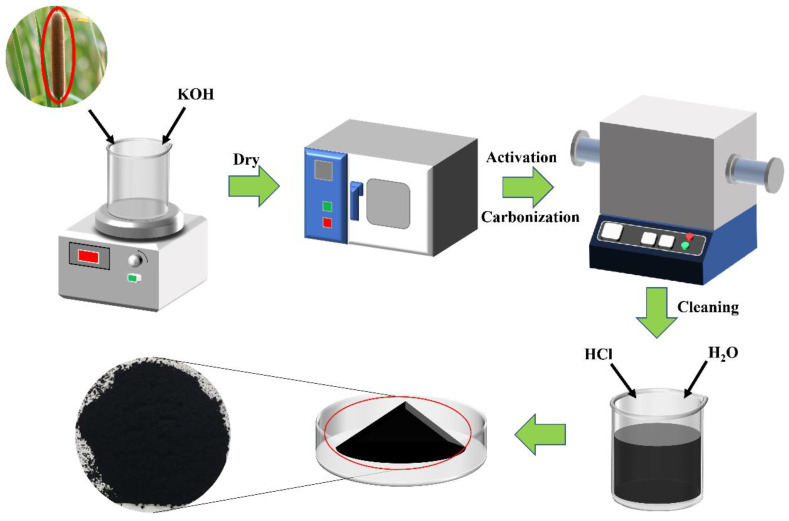
Preparation process of cattail-grass-derived carbons.

The sample preparation process is illustrated in Figure 8. Cattail grass fluff was collected from the flower spike, mixed with KOH activator in a mass ratio of 1:3, and magnetically stirred for 24 h. Then, the samples were dried in an oven. The dried samples were placed in a vacuum tube furnace under an Ar atmosphere (99.99%) at 800 °C for 0.5 h, 1 h, and 1.5 h, respectively, and were denoted as cattail grass with activation treatment (CGA)-0.5, CGA-1, and CGA-1.5. After that, the samples were washed with 1 mol L^−1^ HCl and distilled water to adjust the pH value to neutral. In addition, a sample of cattail grass fluff was also prepared at 800 °C for 1 h. This sample was used as a control group without KOH and was named CG-1.

### 3.2. Material Characterizations

The structure of the samples was characterized by X-ray diffraction (XRD, D/MAX2500PC, Japan) with Cu Kα rays (λ = 0.15406 nm). Raman spectra were obtained using a DXR 03,040,404 (US) Raman spectrometer using a 532 nm argon laser light source at room temperature. A scanning electron microscope (SEM, JEM-2800F, JEOL CO., Akishima, Japan) was used to observe the morphology of the materials. The surface area and pore size distribution were measured at 77 k with a QUADRASORB evo (US) nitrogen adsorption instrument. The chemical structures and functional groups of the samples were investigated using X-ray photoelectron spectroscopy (XPS, Axis Supra, Kratos, UK). The Al Kα radiation of the X-ray source was hν = 1486.6 eV. Infrared spectra (IR) were collected using a Nicolet iS50 (US) Fourier transform infrared spectrometer.

### 3.3. Electrochemical Measurement

The electrochemical measurement was performed on a CR2032 coin-type cell. The reaction mechanism between the electrode and the lithium-ion cell was: xLi + xe^−^ + 6C → Li_x_C_6_. To prepare the working electrode, carbon materials, acetylene black, and polyvinylidene fluoride (PVDF) were mixed at a mass ratio of 8:1:1. N-methyl-2-pyrrolidone (NMP) was added to the mixture as a blending solvent and then stirred evenly. The slurry was coated onto copper foil and dried at 80 °C for 12 h. The mass loading of active material for each electrode was 0.6~0.8 mg cm^−1^. The electrolyte was 1 M LiPF_6_ dissolved in a mixture of ethyl methyl carbonate (EMC) and dimethyl carbonate (DMC). Lithium metal was the counter electrode. The entire installation of coin cells was assembled in an Ar-protected glove box. Cyclic voltammetry (CV) and electrochemical impedance spectroscopy (EIS) measurements were performed using an electrochemistry workstation (CHI660e, Chenhua, Shanghai), where the former was collected at a scan rate of 0.5 mV s^−1^ at 0.02~3 V and the latter with a frequency ranging from 0.01 Hz to 100 KHz. The galvanostatic charge–discharge (GCD) tests were carried out on a LAND battery measurement system (CT3001A, LAND Electronic Co. Ltd., Wuhan, China) at 0.02~3 V. All electrochemical measurements were performed at room temperature.

## 4. Conclusions

A high-performance porous disordered carbon was prepared from cattail grass activated with KOH. Treatment time is a key factor in the modification of structure and morphology. With increasing treatment time, the degree of disorder and specific surface area were enhanced. The resulting CGA-1 shows excellent electronic performance due to its high specific surface area and multi-functional groups. It exhibits a highly reversible specific capacity of 814.7 mAh g^−1^ at a current density of 0.1 A g^−1^ after 400 cycles, which has considerable potential for anode materials in high-performance LIBs.

## Figures and Tables

**Table 1:** Structural parameters of the samples.

Sample	d_002_ (nm)	L (nm)	I_D_/I_G_	S_BET_ (m^2^ g^−1^)	V_micro_ (cm^3^ g^−1^)	V_meso and macro_ (cm^3^ g^−1^)
CG-1	0.374	1.058	0.577	1.175	0.000	0.010
CGA-0.5	0.391	0.665	0.866	886.472	0.414	0.110
CGA-1	0.392	0.671	0.889	714.808	0.322	0.152
CGA-1.5	0.394	0.696	1.056	755.076	0.359	0.190

## Data Availability

Data is contained within the article or Supplementary Material.

## Supplementary Materials

The following supporting information can be downloaded at: https://www.mdpi.com/article/10.3390/molecules28114427/s1, Figure S1: (a) Isotherm, (b) Micropore distribution, and (c) Mesoporous and Macropores distribution of CG–1, CGA–0.5, CGA–1, and CGA–1.5.; Figure S2: Describe the structure changes of (a) CG–1, (b) CGA–0.5, (c) CGA–1, and (d) CGA–1.5; Figure S3: (a) EIS spectra and the simulation circuit of CG–1, CGA–0.5, CGA–1, and CGA–1.5. (b) Equivalent circuit diagram of EIS spectrum; Figure S4: AC impedance spectra of CGA-1 sample before and after 400 cycles; Figure S5: Cycling performance of (a) CG–1; (b) CGA–0.5; (c) CGA–1; (d) CGA–1; at the current density of 0.1 A g^−1^ in LIBs.

## Sample Availability

Samples of the cattail-grass-derived porous carbon are available from the authors.
