# Supplementary material for: Cattail-Grass-Derived Porous Carbon as High-Capacity Anode Material for Li-Ion Batteries
Source: Molecules. 2023 May 29;28(11):4427. doi: 10.3390/molecules28114427 (PMC10254429; doi:10.3390/molecules28114427)
Supplement: Supplementary file 1 [file molecules-28-04427-s001.zip › molecules-2337756-supplementary.pdf]

Supporting Information

Cattail Grass derived Porous Carbon as High-Capacity Anode Materials for Li-ion Batteries

Hui Li [1], Lingyue Song [1], Dongxing Huo [2,*], Yu Yang [3] and Ning Zhang [1] and Jinglong Liang [1,*]

1 Key Laboratory of Modern Metallurgical Technology, Ministry of Education, College of Metallurgy and Energy, North China University of Science and Technology, Tangshan 063210, China

2 College of Mechanical Engineering, North China University of Science and Technology, Tangshan 063210, China

3 Comprehensive Test and Analysis Center, North China University of Science and Technology, Tangshan 063210, China

* Correspondence: huodongxing@ncst.edu.cn (D.H.); ljl@ncst.edu.cn (J.L.)

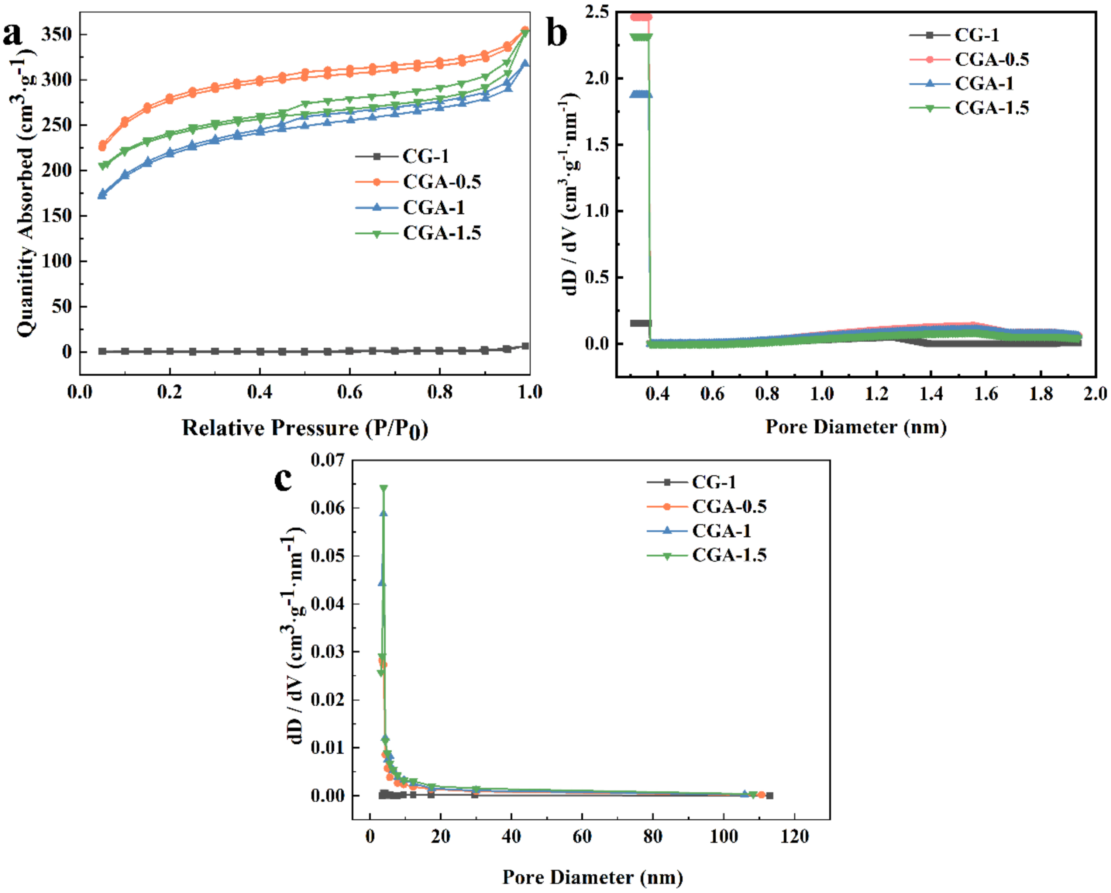

Figure S1. (a) Isotherm, (b) Micropore distribution, and (c) Mesoporous and Macropores distribution of CG-1, CGA-0.5, CGA-1, and CGA-1.5.

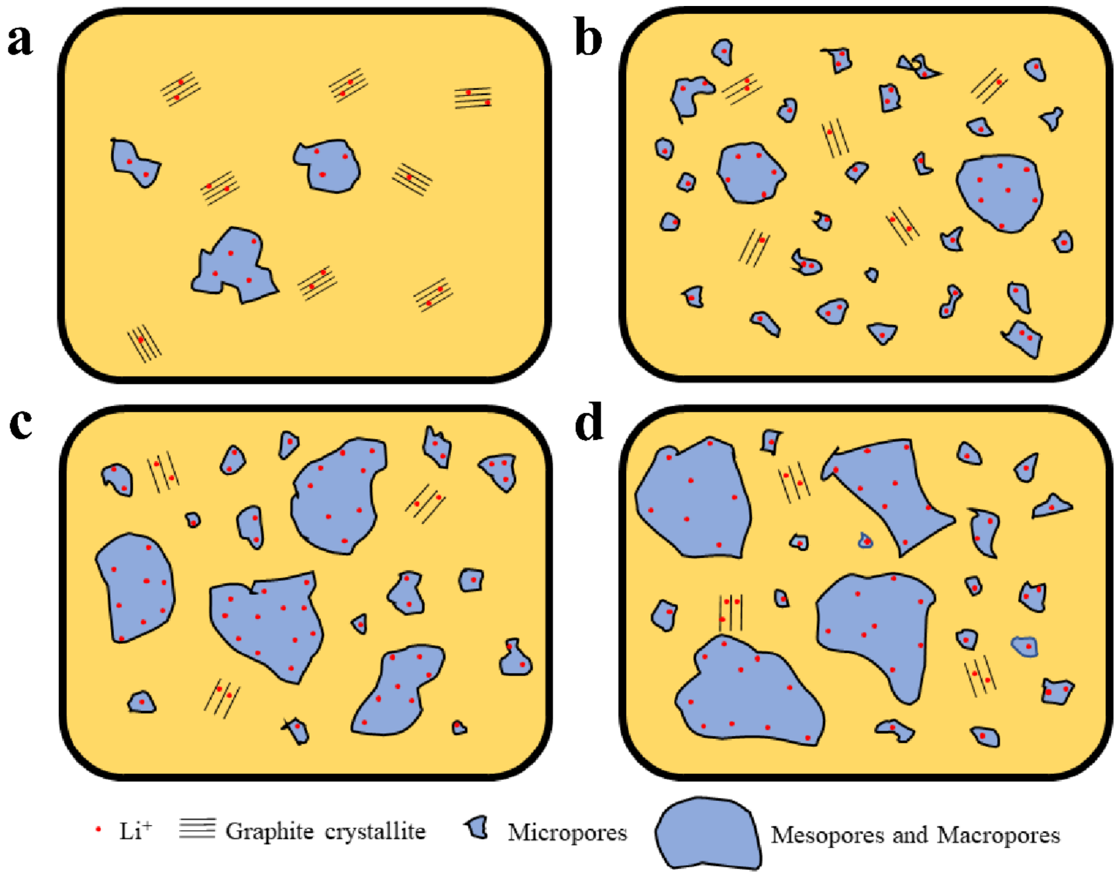

Figure S2. Describe the structure changes of (a) CG-1, (b) CGA-0.5, (c) CGA-1, and (d) CGA-1.5.

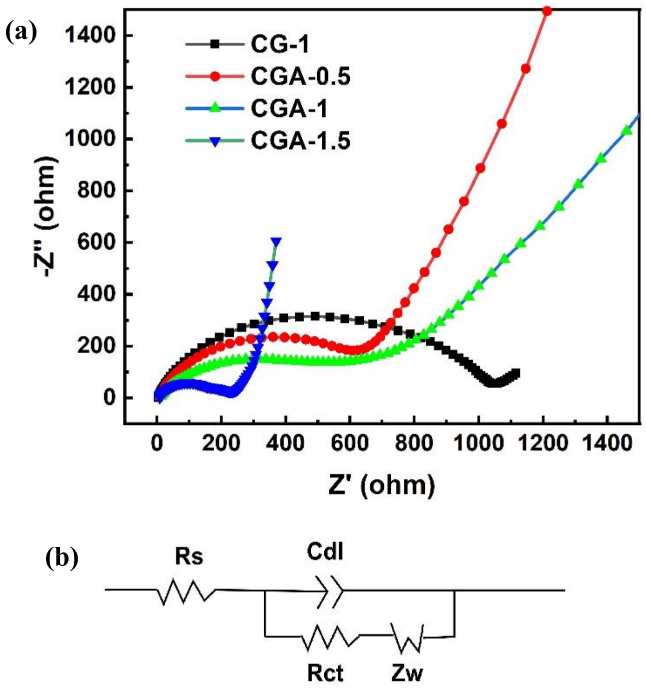

Figure S3. (a) EIS spectra and the simulation circuit of CG-1, CGA-0.5, CGA-1, and CGA-1.5. (b) Equivalent circuit diagram of EIS spectrum.

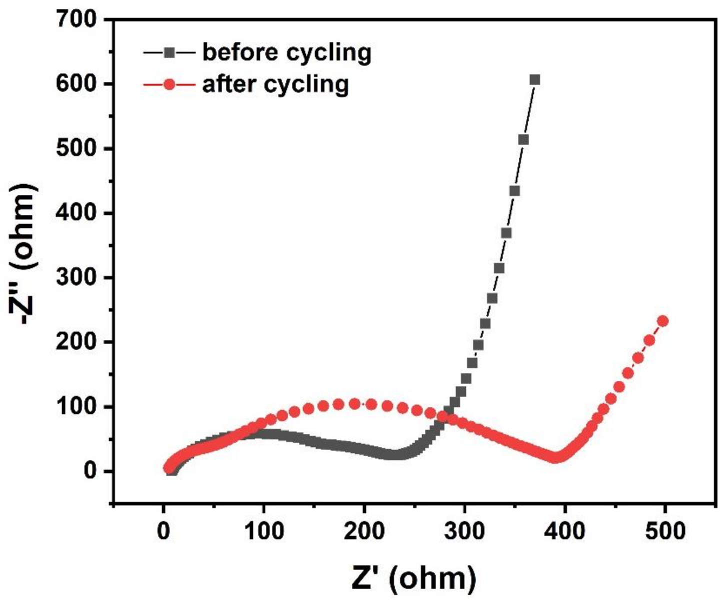

Figure S4. AC impedance spectra of CGA-1 sample before and after 400 cycles.

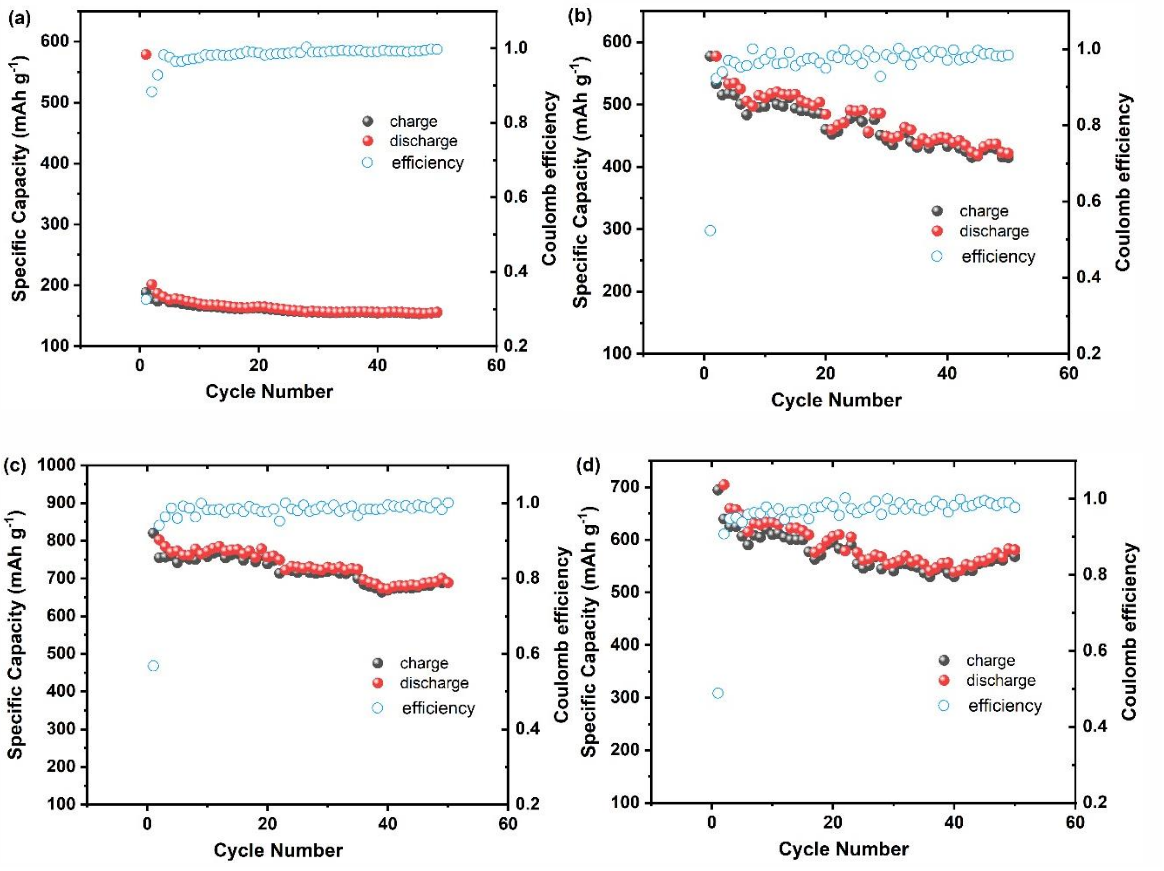

Figure S5. Cycling performance of (a) CG-1; (b) CGA-0.5; (c) CGA-1; (d) CGA-1; at the current density of 0.1 A g^{-1} in LIBs.
